# Death in a Dental Office

**Published:** 1887-09

**Authors:** 


DEATH IN A DENTAL OFFICE.—For the past week, Mr. Hoffman has been suffering with neuralgic pains in the jaw. Thinking that this was due to several decayed teeth, he determined to have them drawn. About 4 30 yesterday afternoon he entered Dr. Melancthon M. Ritchie's office and requested that gentlemen to "pull the aching tooth." Mr. Hoffman at the same time asked the doctor to "give him something," saying that he dreaded "tooth pulling." Dr. Ritchie examined his mouth and told him that he did not think it necessary to administer an anæsthetic, but Mr. Hoffman insisted that he could not stand the operation without it. Dr. Ritchie then acceeded to his request and administered an anæsthetic, compounded as follows: Sulphuric ether two parts, and one part chloroform. As near as could be found out, the dose given was about one ounce. Mr. Hoffman yielded readily to the anæsthetic and two teeth and one root were extracted when the doctor noticed symptoms of collapse. He immediately applied all known resuscitants and sent to a neighboring druggist for aid. He first called on Dr. C. A. Rahter, but found that he was not in his office. He then called upon Drs. I. Elmer Cook, Fred. Coover and P. C. Snyder. These gentlemen repaired to Dr. Ritchie's office at once and did everything within their knowledge to resusciate the patient. All remedies known to the science were applied, but failed to save him from the collapse into which he had fallen, and he expired on the chair to which he had gone for relief. Thus a life that cheered the homes of brother, sister and friend, was snapped off in its very prime, and what was yesterday bright with smiles and sunshine, is to day clouded with sorrow and gloom.

When a *Call* reporter called upon Dr. Ritchie, he found him much distressed over the sad affair, but perfectly willing to give the details.

"Doctor; I feel sorry that this has occurred, but I want to print a true story and beg you will pardon me if I press you close with questions."

The doctor replied, "I am only too glad to give you just what occurred. It is a most lamentable thing for me and I am only thankful that my position may be fairly put before the public."

What did you administer, doctor, and what were the primary symptoms you noticed," said the pardonable inquisitive reporter.

"Well," said the doctor, "to do myself justice, I should say that Mr. Hoffman came to me suffering with acute toothache. He said that he had suffered so much that he could not attend to his duties, and wanted the troublesome member taken out. He insisted, however, that he could not stand the shock unless he could have 'something.' I advised him that the tooth could be removed without an anæsthetic, but he insisted that I should 'give him something.' I then concluded to administer an anæsthetic. I gave him a compound of two parts of sulphuric ether and one part chloroform, and had only given him about one ounce. He yielded readily to the drugs and I extracted two teeth and one root. There were two roots yet remaining which I desired to extract, but after getting rid of those spoken of he sat upright and expectorated. Then he laid back and I thought he hadn't enough and was about to administer more of the compound when I observed such symptoms as led me to desist. His face was overspread with palor and he bore positive signs of exhaustion. This, of course alarmed me, and I took immediate steps to resuscitate him. At this time he was in a semi-recumbent chair. His breathing became irregular and spasmodic. He showed such alarming symptoms that I at once called to my aid such resuscitants as are most generally resorted to under like circumstances. I laid him in a recumbent position and for a moment he seemed to revive, but did not open his lips. I called in my neighbor, William Roe, the druggist, and asked him to supply me with such resuscitants as I might need. I tried artificial respiration, dashing the face with water, and slapping the chest with a wet towel. I first sent young Mr. Roe for Dr. C. A. Rather, but he was not at home. Dr. I. Elmer Cook, Dr. Fred. Coover and Dr. Snyder, however, came to my assistance, and were with me when Mr. Hoffman died. I think the real cause of his death was some trouble on the brain. The condition of the heart was good throughout. His heart beat at least forty minutes after the first inhalation. To my best judgement there was nothing wrong with his heart."

"Did you ever treat him before?" querried the *Call* representative.

"No, I never did."

"Have you administered ether or chloroform or both, frequently?"

"Yes," replied the doctor, "I have frequently given from three to six times the quantity in my practice without ever before experiencing any bad results. You see I used to practice in Boiling Springs and Carlisle, and there one must do all kinds of work. So-to-speak the family physician is dentist, surgeon and physician. I am satisfied that the closest examination will vindicate me from all the abnoxious and disagreeable features of this case.

---

### Monthly Summary.

